# Mobile Software as a Medical Device (SaMD) for the Treatment of Epilepsy: Development of Digital Therapeutics Comprising Behavioral and Music-Based Interventions for Neurological Disorders

**DOI:** 10.3389/fnhum.2018.00171

**Published:** 2018-05-01

**Authors:** Pegah Afra, Carol S. Bruggers, Matthew Sweney, Lilly Fagatele, Fareeha Alavi, Michael Greenwald, Merodean Huntsman, Khanhly Nguyen, Jeremiah K. Jones, David Shantz, Grzegorz Bulaj

**Affiliations:** ^1^Department of Neurology, University of Utah, Salt Lake City, UT, United States; ^2^Department of Neurology, Weill Cornell Medicine, Cornell University, New York, NY, United States; ^3^Department of Pediatrics, University of Utah, Salt Lake City, UT, United States; ^4^Department of Medicinal Chemistry, College of Pharmacy, University of Utah, Salt Lake City, UT, United States; ^5^Software Development Center, University of Utah, Salt Lake City, UT, United States; ^6^Wild Out West, San Rafael, CA, United States

**Keywords:** digital medicine, eHealth, mHealth, refractory epilepsy, antiepileptic drugs, anxiety, antiseizure, Mozart

## Abstract

Digital health technologies for people with epilepsy (PWE) include internet-based resources and mobile apps for seizure management. Since non-pharmacological interventions, such as listening to specific Mozart's compositions, cognitive therapy, psychosocial and educational interventions were shown to reduce epileptic seizures, these modalities can be integrated into mobile software and delivered by mobile medical apps as digital therapeutics. Herein, we describe: (1) a survey study among PWE about preferences to use mobile software for seizure control, (2) a rationale for developing digital therapies for epilepsy, (3) creation of proof-of-concept mobile software intended for use as an adjunct digital therapeutic to reduce seizures, and (4) broader applications of digital therapeutics for the treatment of epilepsy and other chronic disorders. A questionnaire was used to survey PWE with respect to preferred features in a mobile app for seizure control. Results from the survey suggested that over 90% of responders would be interested in using a mobile app to manage their seizures, while 75% were interested in listening to specific music that can reduce seizures. To define digital therapeutic for the treatment of epilepsy, we designed and created a proof-of-concept mobile software providing digital content intended to reduce seizures. The rationale for all components of such digital therapeutic is described. The resulting web-based app delivered a combination of epilepsy self-care, behavioral interventions, medication reminders and the antiseizure music, such as the Mozart's sonata K.448. To improve long-term patient engagement, integration of mobile medical app with music and multimedia streaming via smartphones, tablets and computers is also discussed. This work aims toward development and regulatory clearance of software as medical device (SaMD) for seizure control, yielding the adjunct digital therapeutic for epilepsy, and subsequently a drug-device combination product together with specific antiseizure medications. Mobile medical apps, music, therapeutic video games and their combinations with prescription medications present new opportunities to integrate pharmacological and non-pharmacological interventions for PWE, as well as those living with other chronic disorders, including depression and pain.

## Introduction

Among people with epilepsy (PWE) and their healthcare providers, there are overlapping needs for better control of: (1) seizures, (2) medication adherence and (3) comorbidities. According to the World Health Organization, there are 50–60 million people living with epilepsy world-wide, and only 70% of them respond to current treatments to control their seizures. In the United States alone, there are an estimated 3.4 million PWE, and approximately 150,000 people are diagnosed with epilepsy each year (Epilepsy Foundation, www.epilepsy.com). Newly-diagnosed patients have approximately 50% chance to become seizure free after taking their first antiseizure medication (Brodie et al., 2012, 2013; Chen et al., 2017). Recent work suggests that even after becoming seizure-free, over 60% of patients who discontinue taking antiseizure drugs experienced at least one relapse over a period of 3 years (Park et al., 2017). The diverse etiologies and complex mechanisms of epileptic seizures pose a challenge to reach long-term seizure freedom in approximately 20–30% of PWE (Golyala and Kwan, 2017; Tang et al., 2017). For patients with refractory epilepsy, possible non-pharmacological options to control seizures include dietary interventions (DeGiorgio et al., 2014; Kim et al., 2016; Martin et al., 2016), implantable neuromodulation devices, or brain surgery. Approximately 30% of PWE do not adhere to medication schedules, leading to decreased seizure control (Ettinger et al., 2009; Malek et al., 2017; O' Rourke and O' Brien, 2017). In addition to seizures, PWE often experience depression and anxiety as comorbidities, requiring additional interventions (Kanner, 2016). Taken together, there are multiple needs to develop new therapies with improved efficacy and clinical outcomes for PWE.

Digital health (also mobile health, mHealth, or eHealth,) is a branch of healthcare that employs internet, digital, and mobile technologies for improving health and/or treating specific medical conditions. Many digital health technologies are focused on wellness and health coaching, or disease self-management. mHealth comprises mobile medical apps which receive clearance (as SaMD) from the US Food and Drug Administration (FDA). Examples of SaMD are mobile medical apps, such as BlueStar® (developed by WellDoc) to improve control of glucose blood levels in patients with type 2 diabetes, or reSET® for substance use (addiction), developed by Pear Therapeutics. Music-based videogame MusicGlove and Jinxtronix neurorehabilitation system are examples of the FDA-cleared stroke therapies. Benefits of digital therapeutics include their ability to integrate patient behavior and lifestyle changes with pharmacotherapy (Bulaj, 2014; Bulaj et al., 2016; Mantani et al., 2017; McKennon et al., 2017).

Digital health technologies for PWE comprise mobile apps and internet-based resources delivering epilepsy self-management content (Pandher and Bhullar, 2014; Escoffery et al., 2018; Le Marne et al., 2018; Page et al., 2018). Survey-based studies suggest that PWE and their caregivers are interested in mHealth, but only a small fraction use mobile apps for seizure management (Liu et al., 2015, 2016; Leenen et al., 2016). Pandher and Bhullar reviewed features of 28 mobile apps for seizure management (Pandher and Bhullar, 2014). Content of mobile apps could be categorized into patient education (e.g., general information, seizure triggers, medications, first aid) and self-monitoring (seizure and medication tracking). The authors found relatively low quality of educational components in the apps, whereas seizure diaries were the most commonly incorporated features (Pandher and Bhullar, 2014). Recently, 20 mobile apps for epilepsy self-management were reviewed with respect to self-management functions and behavior change strategies, as well as evaluated for their engagement, functionality, esthetics, information quality and satisfaction using the Mobile App Rating Scale (MARS) (Escoffery et al., 2018). An example of online self-management platform for epilepsy patients is WebEase (Epilepsy Awareness, Support and Education) delivering three modules focused on medications, stress and sleep (DiIorio et al., 2009a,b). Another web-based intervention is the Epilepsy Journey, targeting executive function deficits in adolescents with epilepsy (Modi et al., 2017). Feasibility of mobile and internet-based delivery of psychosocial interventions and cognitive behavioral therapy for PWE further emphasizes opportunities for digital interventions (Hixson et al., 2015; Gandy et al., 2016; Glynn et al., 2016). Wearables intended to detect and predict seizures have been developed, for example SmartWatch and the FDA-cleared Embrace by Empatica. To the best of our knowledge there are no published studies on effects of digital health technologies on reduction of seizure frequency.

For PWE, digital health technologies offer opportunities to improve therapy outcomes by integrating prescription medications with specific musical compositions (Bulaj, 2014). As summarized in Table 1 and described in the Results section, there are several published reports on antiseizure effects of Mozart's sonata K.448 on reducing seizure frequencies and epileptiform discharges in PWE. Antiseizure effects of K.448 were observed in children with refractory epilepsy (Lin et al., 2011a), and in those after first unprovoked seizures (Lin et al., 2014a). The clinical findings are also supported by animal studies suggesting that Mozart's K.448 can reduce seizure frequencies in rats (Lin et al., 2013), upregulates expression of brain-derived neurotrophic factor (BDNF) in rats (Xing et al., 2016a,b,c), reduces cognitive impairment in status epilepticus rats (Xing et al., 2016b), increases brain levels of dopamine in rats (Tasset et al., 2012), and modulates expression of several genes involved in neurotransmission in the hippocampus and the forebrain cortex in mice (Meng et al., 2009). The possible mechanism by which music exerts antiseizure effects may include neuromodulation of the parasympathetic system (Lin et al., 2013; Dastgheib et al., 2014).

**Table 1:** A summary of clinical studies of listening to specific Mozart's compositions on epileptic seizures and epileptiform discharges.

**Clinical study**	**Main outcome**
Hughes et al., 1998	Mozart's music significantly reduced epileptiform activity in epilepsy patients, including those with status epilepticus
Hughes, 2001	Mozart music decreased epileptiform activity in adult patients with status epilepticus
Lin et al., 2010	Piano K.448, but not string K.448, reduced epileptiform discharges in epilepsy patients
Lin et al., 2011a	Mozart's music significantly (>50%) reduced seizure frequency in pediatric patients with refractory epilepsy
Lin et al., 2011b	1-, 2-, 6-months of listening to Mozart significantly reduced epileptiform discharges in children taking antiepileptic drugs
Bodner et al., 2012	Mozart's music significantly reduced seizures in adult epilepsy patients
Lin et al., 2012	Two Mozart sonatas K.545 and K.448 were effective in reducing epileptiform discharges in the brain in epilepsy patients
Lin et al., 2013	Listening to Mozart reduces the heart rate and activates the parasympathetic nervous system
Lin et al., 2014a	Seizure recurrence rate was significantly lower for the treatment group (listening to K.448 for 10 min before bedtime) than the control
Lin et al., 2014b	EEG can be used to predict beneficial effects of the Mozart's music for epilepsy patients
Coppola et al., 2015	15-day treatment with the Mozart's music resulted in >50% reduction of seizures in 45% of children with epileptic encephalopathy
D'Alessandro et al., 2017	Once daily listening to Mozart K.448 for 6 months yielded a 20% reduction in seizure frequency
Coppola et al., 2018	Listening to Mozart K.448 and other Mozart compositions for 2 weeks (2 h/day) resulted in 37–77% reduction of seizures in children with epileptic encephalopathy

**Figure 1:**
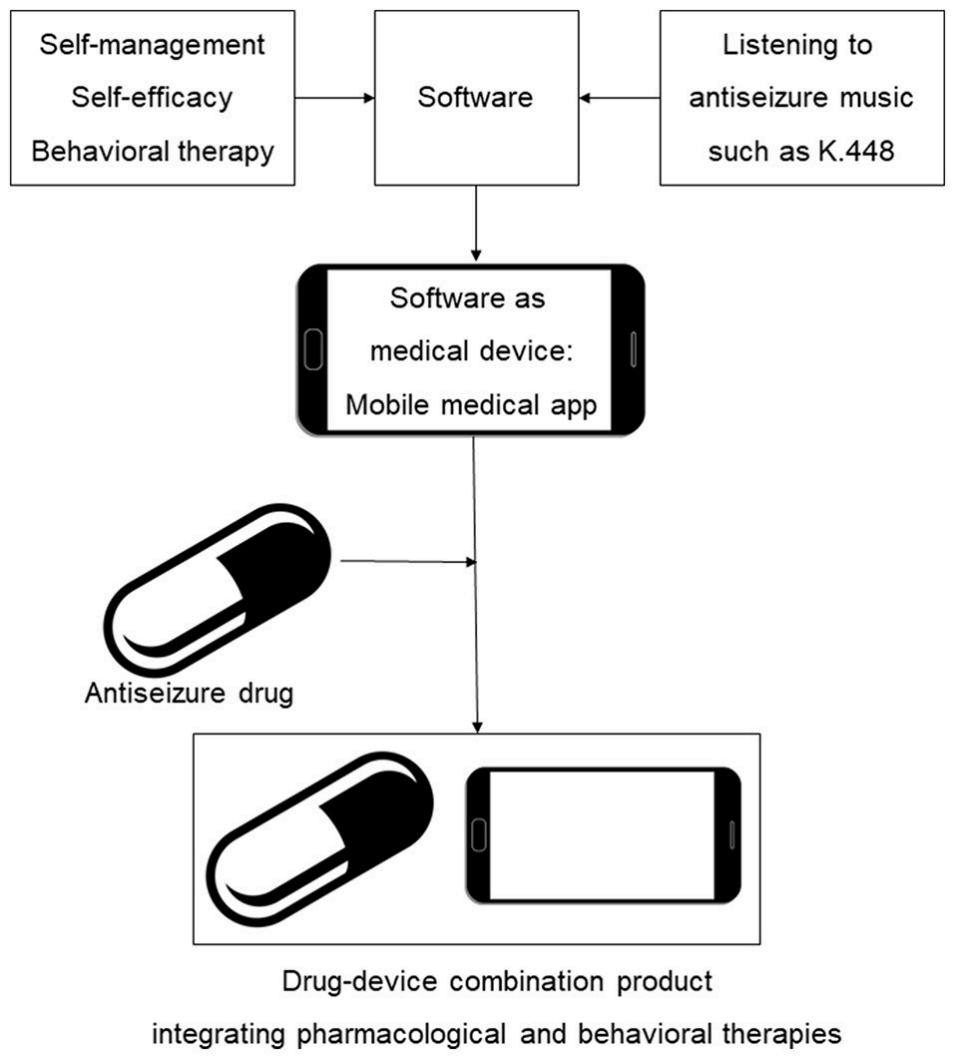
Scheme illustrating integration of behavioral interventions, self-care (self-management and self-efficacy) and antiseizure music into mobile medical app (SaMD) and a drug-device combination product for epilepsy. An incentive for clinical development and regulatory approval of such digital therapeutics and their combinations with brand and generic drugs is the potential for long-lasting intellectual property protection based on copyrights of creative works and software (Bulaj, 2014; Bulaj et al., 2016).

In this work, we describe initial steps in development of digital therapeutics for epilepsy, including the survey-based study and design of prototype mobile software intended to reduce seizures. We provide a rationale for mobile medical app content which integrates antiseizure music, epilepsy self-care and elements of cognitive behavioral therapy. As illustrated in Figure 1, our long term goal is to develop SaMD as adjunct digital therapeutic for the treatment of epilepsy, followed by integration of specific antiseizure drugs with SaMD, yielding, from a regulatory perspective, a drug-device combination product. The drug-SaMD combination product offers means to: (1) integrate pharmacological and behavioral therapies with self-care, (2) create new personalized treatments for epilepsy, and (3) improve medication adherence and patient engagement. Our work has implications beyond epilepsy, since combining prescription medications and music-based interventions is also applicable to the treatment of depression (Schriewer and Bulaj, 2016), pain (Chai et al., 2017) and potentially other neurological disorders (Sihvonen et al., 2017).

## Methods

### The survey study

The study was conducted in the University of Utah Adult Comprehensive Epilepsy Clinic. The institutional review board (IRB) at University of Utah reviewed and approved the study. The study was determined to be “exempt” by the IRB due to its minimal risk to participants. The IRB approved an authorization cover letter for participants to sign prior to completing the study questionnaire. The study information was kept in secured manner and electronic records of patients were password protected. The questionnaire was designed and consisted of seven questions related to preferences in using mobile apps for seizure management and control (Supplementary Information). The questionnaire collection forms were created for each participant and information was directly captured in the electronic clinical data management tool REDCap (Research Electronic Data Capture Software). This study was conducted between months June and August 2017, and involved PWE aged 18 years and older who were regular smartphone users. Patients without access to smartphones, and/or who were cognitively impaired were excluded from the study population. A total of 40 individuals participated in this study.

After signing consent, each participant was first provided with brief introduction about the purpose of this study. Any person who did not agree to participate in this study was excluded. Participant's protected health information (PHI) was linked to their questionnaire to avoid data duplication for which the IRB has approved an authorization cover letter for participants to sign prior to enrollment. Questions were explained to the participants by the moderator, who was a student trained in qualitative research methods. The epilepsy attending and/or study coordinator and/or a student were present during data collection. The participants were asked to select the options that they felt were most beneficial and desirable for them, and features they wanted in the mobile app. The questionnaire answers were completed by the participants using the REDCap. While the authors had a positive attitude toward mobile app, they strived to remain neutral in conversation with the participants. Data analysis and reports were viewed on REDCap Stats and Charts.

### Design of mobile software prototype

Using PubMed, literature search was performed to identify clinical studies of non-pharmacological interventions that reported seizure reduction in PWE. The following key words were used alone and in combination with “epilepsy” or “epileptic seizures”: self-management, self-efficacy, self-care, psychosocial, cognitive behavioral therapy, educational intervention, web-based intervention, internet, music, Mozart. Information from the published studies reporting a reduction of seizure frequency was analyzed and incorporated into software content. When designing the prototype mobile software we hypothesized that the duration of the digital therapy would last preferably 1 year, or longer, during which time a patient would be engaged with the mobile app for approximately 10 min daily. Therefore, user experience (UX) interface, interactivity, gamification and novel daily content were important factors to maximize patient's engagement. To create a prototype of the mobile software, a web-based version was built using HTML5 and hosted on a shared-server Linux platform. Visual displays of the digital content were discussed among software and UX engineers and clinical team members.

## Results

### The survey study

The main purpose of our survey study was to evaluate the patient's interest in using a mobile app for seizure control and self-care. Our questionnaire was focused on questions about preferred features in a mobile app related to seizure management, wellbeing, self-awareness, empowerment, and engagement in the therapy. All questions and answers are provided in the Supplementary Information. A total of 40 individuals participated in this study. The survey results show that over 90% of patients were interested in using a mobile app to help manage their seizures. In relation to monitoring epilepsy, 85% of patients were interested in diary to record date of their seizures, 73% were interested in recording type of their seizures, 78% were interested in logging the missed dosages of their medications. Regarding automated reminders, a majority was interested in reminders to keep their follow up appointments (80%), to refill their medications (73%), to take their medication on time (68%).

**Figure 2:**
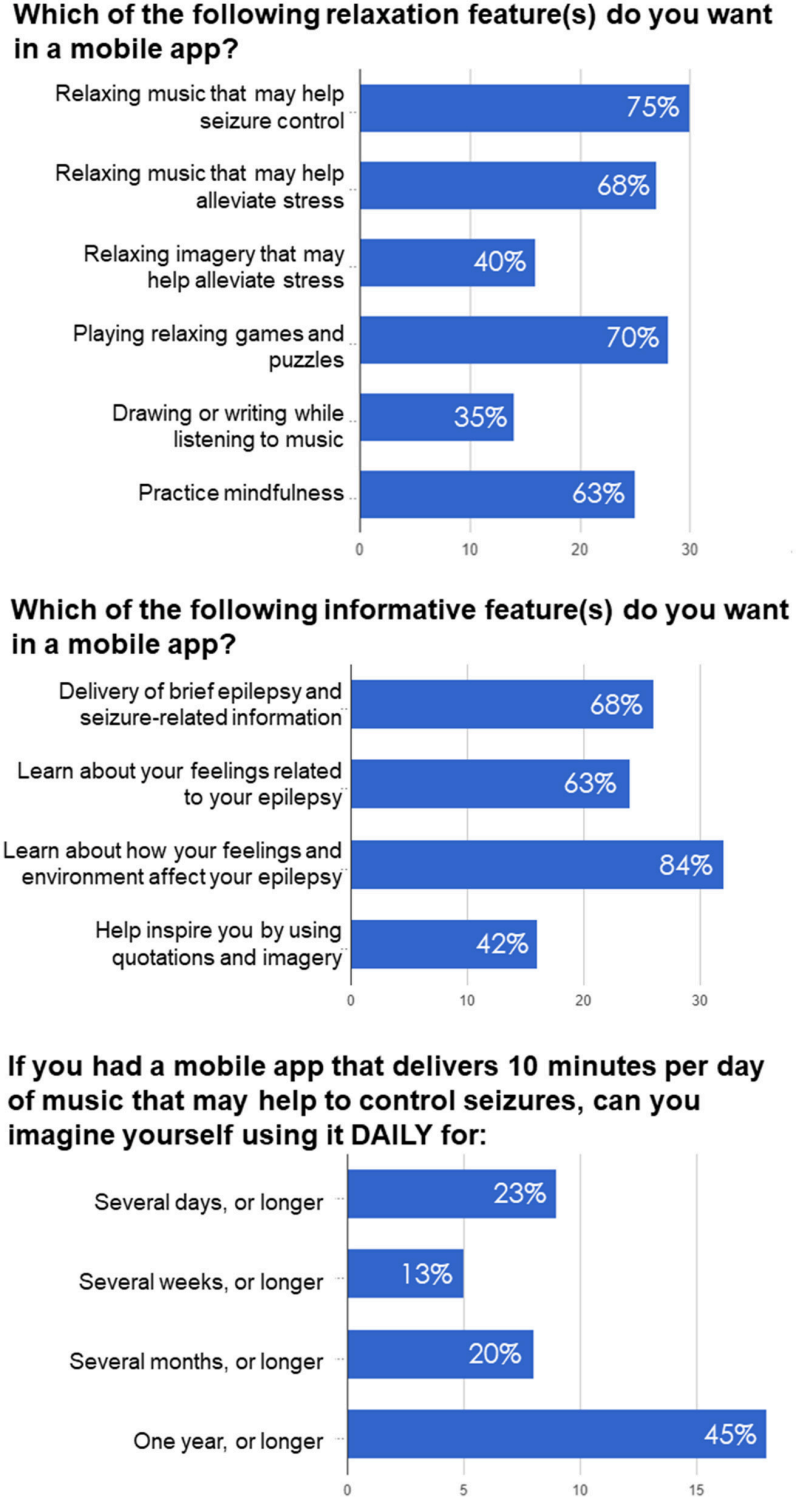
Examples of questions and responses in the survey-based study examining preferences of PWE regarding antiseizure music-based intervention, self-awareness and self-care delivered via mobile app.

In relation to understanding their disease, 68% of responders were interested in delivery of brief epilepsy and seizure-related information (Figure 2), 84% were interested about how their feelings and environment affect their epilepsy, 63% were interested in learning about feelings related to their epilepsy, while 42% were interested in being inspired by quotations and imagery. Additionally, 5% of participants did not find informative features applicable to them and therefore left this section blank.

Two questions related to music-based intervention were focused on seizure control and comparing music with other relaxation techniques like imagery, games and puzzles, and practicing mindfulness. About 75% of responders were interested in music that may help seizure control and 68% were interested in relaxing music that may help alleviate stress, while 40% of patients were interested in relaxing imagery that may help alleviate stress (Figure 2). 70% of patients were interested in playing relaxing games and puzzles, while 35% were interested in drawing or writing while listening to music. About 63% were interested in practicing mindfulness.

To determine potential adherence to music-based intervention, we asked participants how long they would be willing to listen to antiseizure music, if it was delivered daily for 10 min. Previous studies on the effects of Mozart music on seizure reduction suggested that music intervention was effective when delivered for at least 1 month, while most studies tested antiseizure effects of music delivered for either 6 or 12 months (Lin et al., 2011a; Bodner et al., 2012; Dastgheib et al., 2014; D'Alessandro et al., 2017). As shown in Figure 2, a majority of responders (65%) imagined using it for several months or longer (including 45% who imagined such therapy lasting for 1 year or longer). 13% of responders could use it for several weeks or longer, while 23% for several days or longer. In summary, the questionnaire responses confirmed PWE's interest in using a mobile app for seizure control, management and self-care.

### A rationale for integrating non-pharmacological interventions into mobile software

PubMed literature search identified non-pharmacological interventions that resulted in reducing the frequency of seizures in PWE (Tables 1, 2). Our hypothesis is that incorporating non-pharmacological interventions and self-care into mobile software can yield digital therapeutics (SaMD) for epilepsy (Figure 1). Clinical studies showed that daily listening to Mozart's sonata K.448 significantly reduced frequency of epileptic seizures and/or epileptiform discharges in adult and pediatric patients with epilepsy (Table 1). Listening to K.448 was effective in reducing seizure frequency even after 1 month, and further improved seizure frequency over 6 month treatment time (Lin et al., 2011a). Studies also showed that additional Mozart's compositions, including K.207, K.218, K.314, K.482, K. 545, and K.551 had positive effects on children with epilepsy, including reduction of epileptiform discharges and reduction of seizure frequency (Lin et al., 2012; Coppola et al., 2015, 2018). Possible mechanisms by which music may exert its antiseizure effect include activation of the parasympathetic system (Lin et al., 2013), stabilization of the hypothalamic-pituitary-adrenal (HPA) axis (Maguire and Salpekar, 2013; Wulsin et al., 2016), or include dopaminergic signaling through D2-like receptors (Salimpoor et al., 2011; Bozzi and Borrelli, 2013).

**Table 2:** A summary of clinical studies showing reduction of seizures following behavioral interventions.

**Study**	**Main outcome**
Gillham, 1990	Significant reduction in seizure frequency in epilepsy patients, during and 6 months after self-management
Tieffenberg et al., 2000	Children with epilepsy had significantly less seizures in the behavioral-educational intervention group
May and Pfafflin, 2002	Educational intervention resulted in significant reduction of seizure frequency in 19% of patients
Lundgren et al., 2006	Behavioral intervention in drug-resistant epilepsy patients resulted in significant reduction in seizure frequency and duration in the treatment group
Lundgren et al., 2008	Behavioral intervention was effective in reducing seizure frequency in epilepsy patients. 50% patients became seizure-free after intervention
McLaughlin and McFarland, 2011	Seizure frequency was significantly reduced (from average 6.33 to 1.39 seizures/month), as compared to control group in epilepsy patients
Tang et al., 2015	Seizure frequency was significantly reduced after 6-week of four biweekly mindfulness-based therapy, as compared to control group (social support)
Haut et al., 2018	Two behavioral interventions, progressive muscle relaxation and focused attention, produced significant reduction of seizures (by 25–29%) in patients with drug-resistant focal seizures

**Figure 3:**
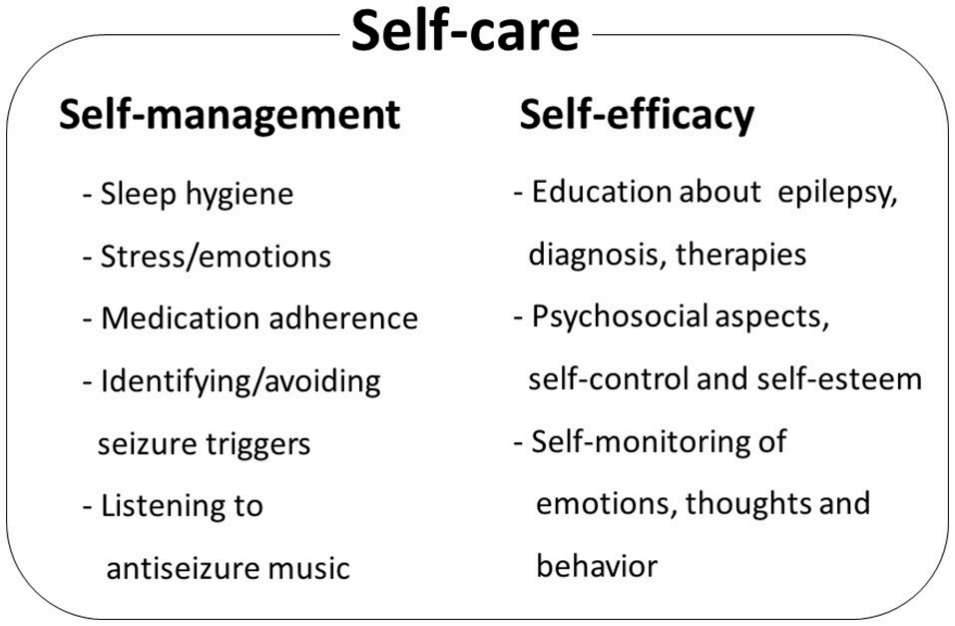
Epilepsy self-care comprises self-management components and patient's self-efficacy. Patient's behaviors and life-style may improve seizure control by identifying and managing seizure triggers. Patient's perceived self-control of epileptic seizures, awareness of behavior and emotional status and self-esteem contribute to health-related locus of control and self-efficacy. Psychosocial and behavioral therapy studies identify key elements that contribute to seizure control. Additional patient behavioral component is daily listening to antiseizure music, such as Mozart's sonata K.448.

Studies of psychosocial interventions, behavioral cognitive therapy and self-management for PWE (Kotwas et al., 2017; Michaelis et al., 2017) suggest that self-care practices may lead to reduction of seizure frequency (Mittan, 2009; Edward et al., 2015; Table 2, Figure 3). In the review of psychosocial interventions in epilepsy (Mittan, 2009), the author found five studies reporting positive findings out of seven studies that measured seizure control. A significant reduction of seizures in older adult patients with epilepsy was observed at 3-month follow up after 6-week cognitive behavioral therapy intervention (McLaughlin and McFarland, 2011). Positive results in seizure reduction were observed in randomized trial of behavioral interventions in patients with the refractory epilepsy (Gillham, 1990). Another randomized trial of behavioral therapy (acceptance and commitment therapy) showed a significant reduction of seizure frequency in patients with the refractory epilepsy (Lundgren et al., 2008). Behavioral and educational interventions were effective in reducing seizures in both pediatric and adult populations (Spector et al., 1999, 2001; Tieffenberg et al., 2000; May and Pfafflin, 2002), also emphasizing importance of self-efficacy, knowledge, understanding and management of emotional health (Spector et al., 2000, 2001; McLaughlin and McFarland, 2011). Noteworthy, targeting the HPA axis for reduction of seizures has been proposed (Maguire and Salpekar, 2013; Wulsin et al., 2016). As summarized in Figure 3, when designing the prototype mobile software for epilepsy patients, we incorporated key behavioral and self-care aspects that support seizure control through a combination of education, awareness and behavioral interventions.

### Design of the prototype mobile software

**Figure 4:**
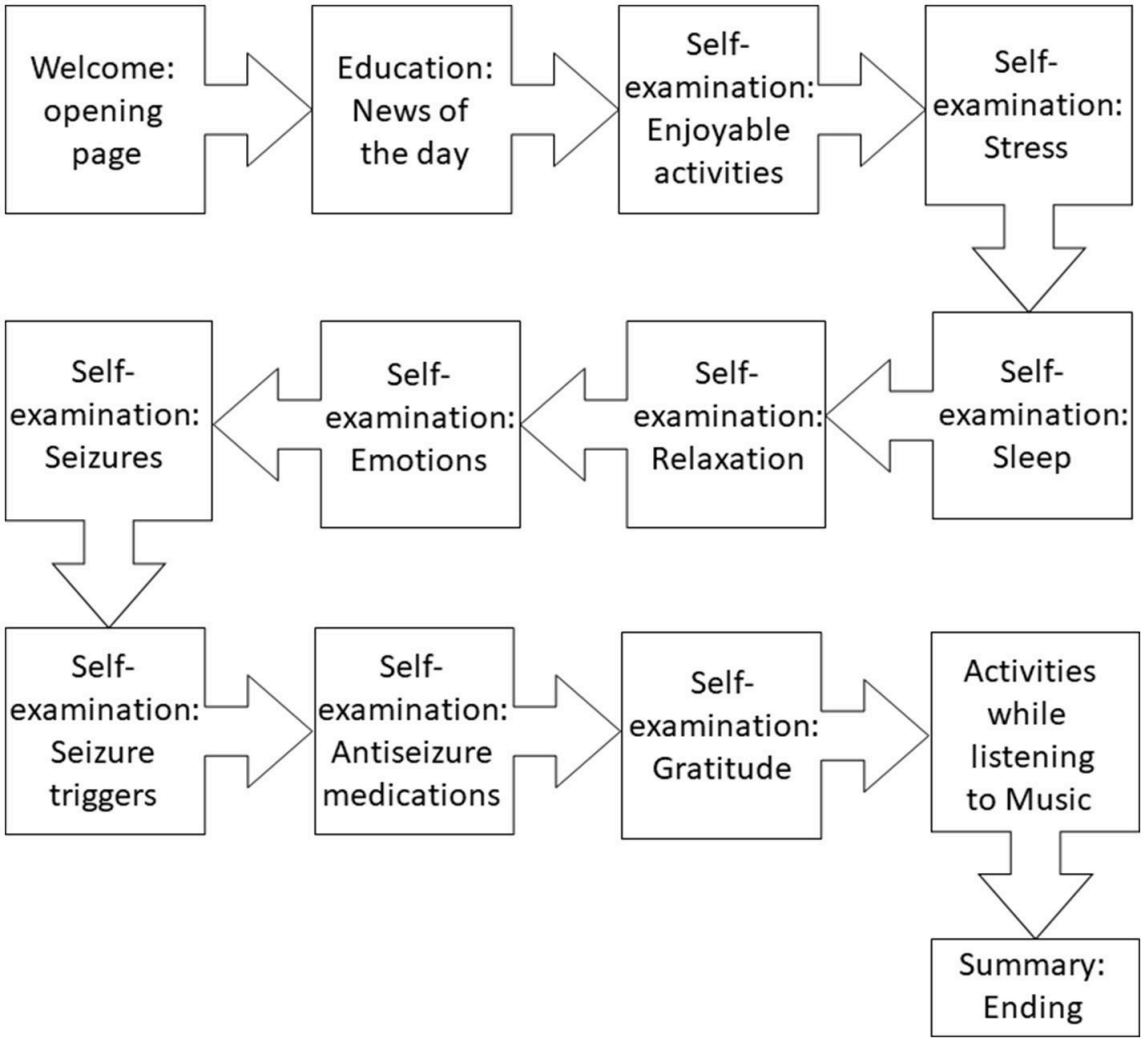
Flow of the proof-of-concept mobile software for epilepsy patients. The interaction between a patient and the software was intended to last 10 min daily.

**Figure 5:**
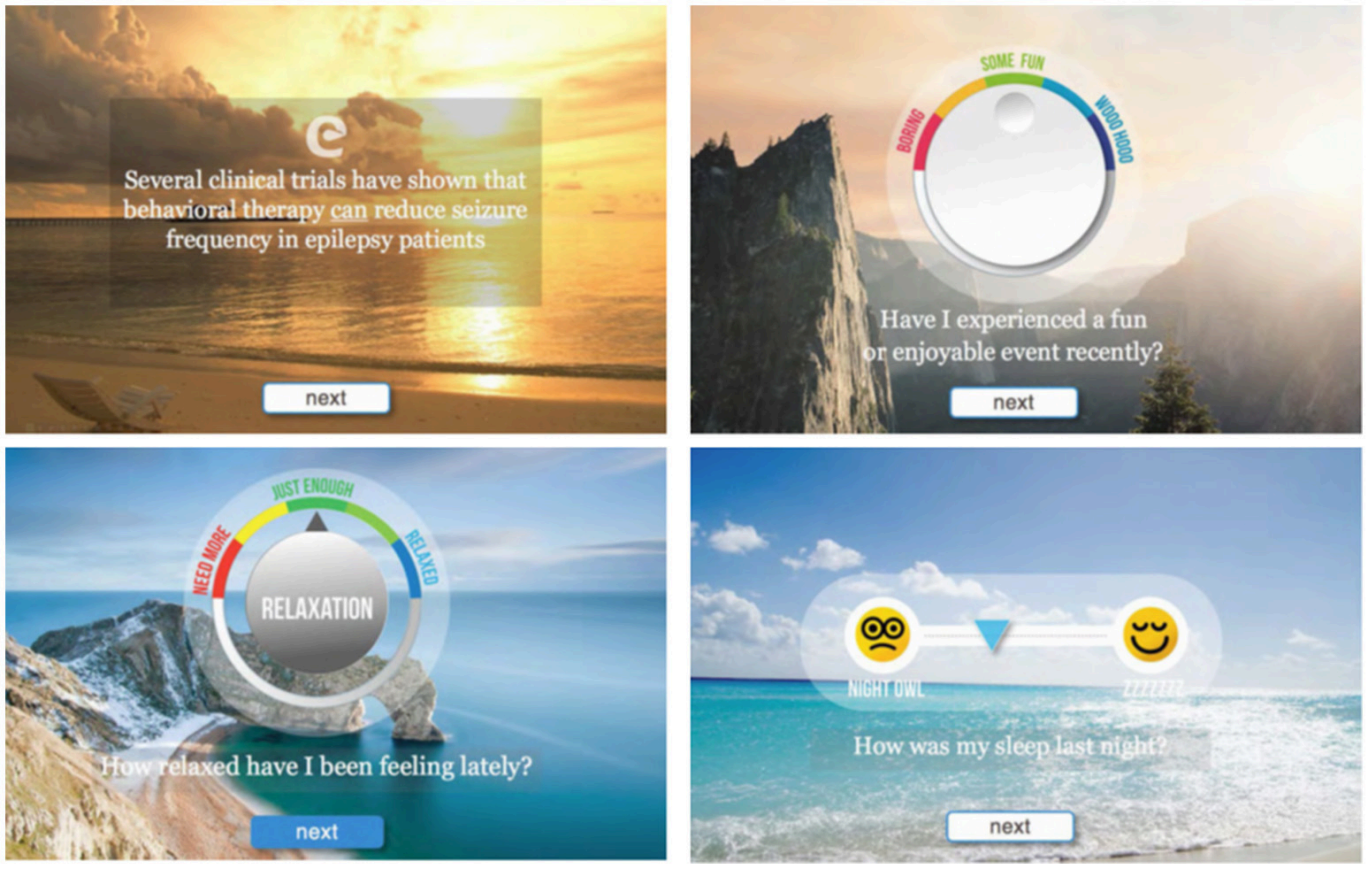
Representative screen shots from the proof-of-concept mobile software for epilepsy patients. Background images are presented as sliders and provide additional 3D perception.

We designed and created the proof-of-concept software, which consisted of two main modules: (1) self-care (education and self-examining), and (2) leisure activities while listening to a total of 10 min of antiseizure music. As shown in Figure 4, key elements of the proof-of-concept mobile software included: (1) welcoming front page, (2) epilepsy-focused educational content, (3) self-management provided as self-examination, (4) improving self-esteem and self-efficacy, (5) listening to antiseizure music, (6) gamification, and (7) summary page. Examples of web-based screens are illustrated in Figure 5. Opening page was designed to welcome a patient while summarizing cumulative “score” of being engaged with the mobile app (Figure 4). Entering next pages automatically started playing antiseizure music, and for the proof-of-concept mobile software we looped the first movement of Mozart's sonata K.448. The next page provided “daily educational information” related to epilepsy and focused on the epilepsy knowledge, therapy, self-care and advances in clinical research. Next several pages were designed as self-examination of self-care components related to seizure control and quality of life (Figure 4). Interactive self-examining was delivered by structuring questions as introspective: “*Have I experienced*….” or “*How was my sleep last night?*” encouraging patient self-reflection before answering these questions. Self-examination was focused on enjoyable activities, relaxation, daily gratitude (reinforcing positive attitude and self-esteem), potential seizure triggers (stress, lack of sleep, emotions, others), and medication compliance reminders. After self-examination, the remaining time (of a total of 10 min) of listening to the antiseizure music could be spent on leisurely or creative activities chosen by the patient.

The prototype mobile application was designed to create an engaging and intuitive user experience. The web-based prototype was built using HTML5 and hosted on a shared-server Linux platform. The interactions were primarily audio and visual, including progress feedback that can be monitored/recorded using emoticon-type symbols as opposed to a more traditional journaling process. As illustrated in Figure 5, the application's user interface was focused on making the experience easy and friendly, with a clean and simple modern interface.

## Discussion

Despite increasing number of digital health technologies for seizure management, we are not aware of development of mobile medical apps intended to reduce frequency of seizures in epilepsy patients. Our survey suggest that about two-thirds of PWE could see themselves using such mobile software for at least several months. Patients preferred automated features for different aspects of seizure and disease management, and were also interested in the relaxation features of the app. One interesting finding was that our subjects' interest was more in passively listening to music than actively using imagery. They were also more interested in playing games or puzzles, as compared to writing or drawing while listening to music. We concluded that in our subject population there was less interest in relaxation techniques that used mental creativity (drawing, writing or using imagery). There was more interest in relaxation techniques consisting of observation (practicing mindfulness) or immersing passively by listening to music, or engaging reactively in puzzles and games, as compared to those techniques involving mental creativity. The reasons for preferences toward more passive activities are not clear at this time, but it may be that such relaxation techniques might increase brain stem parasympathetic activity, which in turn may have some anti-seizure effects. Bodner and colleagues reported that antiseizure music was also effective in reducing seizures when delivered during sleeping (Bodner et al., 2012), suggesting that for PWE who favor less engagement there are opportunities to develop more passive strategies (like streaming antiseizure music during sleep).

Music is a non-pharmacological modality that produces pleiotropic physiological effects, including activation of the dopaminergic system and D2 receptors (Salimpoor et al., 2011). Music-evoked neurochemical changes in the brain suggest therapeutic potential in affective and neurological disorders (Koelsch, 2010, 2014; Chanda and Levitin, 2013). Increasing evidence from preclinical and clinical studies supports the development of digital therapeutics for epilepsy delivering specific musical compositions. However, listening daily to the same music (e.g., Mozart's K.448) for several months may produce undesirable and even maladaptive effects including a lack of interest, irritation and “desensitization” of physiological effects. Results from a recent animal study suggest that the CNS effects of K.448 may be in part due to the so called Mozart rhythm effect (Xing et al., 2016c). To identify musical compositions similar to Mozart's sonata K.448, a neurologist/clinical neurophysiologist JR Hughes used computer analysis of periodicity and melodic lines in 330 compositions of Mozart, 155 of JS Bach, 61 of Beethoven, 58 of Chopin and 23 of Wagner, and selected 25 compositions with the highest values of long-lasting periodicities (Hughes, 2001, 2002), expanding a potential repertoire of music for clinical testing for antiseizure properties.

Based on our observations and other studies (van Andel et al., 2011; Walker et al., 2012, 2014), it is apparent that in addition to PWE, their caregivers and close friends may also benefit from using mobile software designed for epilepsy self-care. Social support needs of PWE and caregivers offer future opportunities to expand the mobile app content in which: (1) PWE and their closest support group can share access to learning about epilepsy, self-management and wellbeing, (2) PWE could connect with their primary caregivers, while still maintaining the autonomy to control their individual use of the app, (3) caregivers could update seizures recordings for those that the patient might not have been aware of, (4) PWE and their loved ones can socially interact hence minimizing the feeling of isolation that is known to often accompany those living with epilepsy.

**Figure 6:**
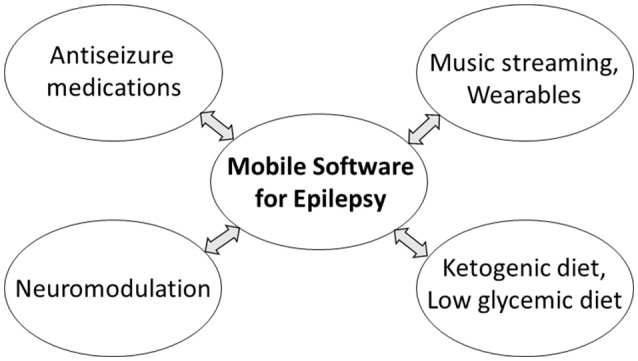
Possible combinations of mobile medical app for the treatment of epilepsy with pharmacological and non-pharmacological interventions. Some combinations may require additional safety and efficacy studies and regulatory authorization before marketing.

Mobile software for epilepsy patients is a non-pharmacological modality that can be easily combined with other therapies (Figure 6). The most apparent combinations are with antiseizure medications (Bulaj, 2014), or with dietary interventions such as ketogenic and low-glycemic diets (DeGiorgio et al., 2014; Kim et al., 2016; Martin et al., 2016). A rationale for combining mobile software with pleiotropic natural products including n-3 polyunsaturated fatty acids was previously discussed (DeGiorgio et al., 2014; Bulaj et al., 2016). For patients with refractory epilepsy, mobile medical app delivering self-care and antiseizure music can be validated for use with neuromodulation devices like vagus nerve and deep brain stimulation, VNS and DBS, respectively, although additional safety and efficacy studies and regulatory authorization would be required before marketing such combination therapies. Recently, we described music streaming as an adjunct digital therapy for depression, anxiety and bipolar spectrum disorders (Schriewer and Bulaj, 2016). Music and multimedia streaming of the antiseizure digital content can complement the use of a mobile medical app for seizure control. In addition to increasing patient engagement, web-based streaming offers an additional safety feature by mitigating potentially stressful events such as losing or breaking a mobile phone, tablet, or laptop that hosts the SaMD. Technological advancements in wearables that provide real-time physiological feedback (e.g., smart watches by Empatica, or mobile EEG systems by Emotiv, Muse, or NeuroSky) may be used to optimize streamed content, similarly to the strategy used for patients with depression (Ramirez et al., 2015). The development of clinically-validated, multimedia streaming with subsequent integration with the mobile app for PWE are longer-term prospects.

In the present study, we describe the initial stage of developing prescription digital therapeutic for the treatment of epilepsy in patients already taking antiseizure medications. Due to multiple challenges in developing such innovative medical technology, we exercise step-wise and iterative approach which started with the survey and the proof-of-concept prototype design (this work). Before any clinical testing, the web-based prototype will be converted into an alpha version of the mobile medical app, due to current regulatory guidelines. The step-wise development plan includes: (1) pilot feasibility study of the alpha version, (2) optimization based on feedback from the pilot study, yielding beta version, (3) a randomized, controlled pivotal (efficacy) trial of the beta version. Since the mobile medical app is intended to be used as add-on digital therapeutic, two main clinical outcomes to be studied will be seizure frequency and medication adherence. For the alpha version of the mobile medical app, the software will be built using best-of-breed software engineering standards and the tools necessary to support highly secure and readily available mobile software. While the primary experience will be native to the mobile device, the software will include a server-side component used to manage software updates and limited user data. The system will comply with all relevant FDA regulations and be HIPAA compliant. Both Android and iOS devices will be included in the initial release. The regulatory pathway will follow the recommendations in the FDA guidance document that can be found at www.fda.gov/MedicalDevices/DigitalHealth/MobileMedicalApplications/default.htm. Anticipated challenges during development include long-term patient engagement, privacy protection and cybersecurity.

This paper also describes the development strategy for merging pharmacological and behavioral therapies for PWE by means of drug-device combination products in which an antiseizure drug works together with a mobile medical app (Figures 1, 6). Companies including Pear Therapeutics and Akili Interactive already develop clinically validated mobile apps and videogames delivering disease-specific behavioral therapy as stand-alone “prescription digital therapeutics,” or to be used with prescription medications. This strategy illustrates broader impact of digital therapeutics allowing integration of self-care and behavioral therapies with pharmaceutical drugs (Bulaj et al., 2016). From a US FDA regulatory perspective, approval of a drug-device combination product incorporating a mobile medical app for use with a drug typically involves first completing a pilot investigation to confirm the feasibility of the approach. After device refinements, a pivotal clinical investigation is performed to reasonably establish the safety and effectiveness of the drug-device combination product. An incentive for innovating therapies using digital therapeutics is their copyright-based intellectual property protection, applicable even to combinations with generic drugs (Bulaj, 2014). Regulatory processes for medical devices vary from country to country, and as digital therapy is a relatively new field, regulatory requirements for developing and obtaining regulatory authorization to market mobile software as a medical device continue to evolve. Challenges associated with developing digital therapeutics include: (1) mitigating the gap between rapidly changing technologies and the slower-pace of clinical development, (2) mitigating patient risks including stress associated with losing/breaking a digital device, (3) incorporating robust cybersecurity measures, (4) ensuring patient engagement, and (5) health care system implementation and reimbursement.

**Figure 7:**
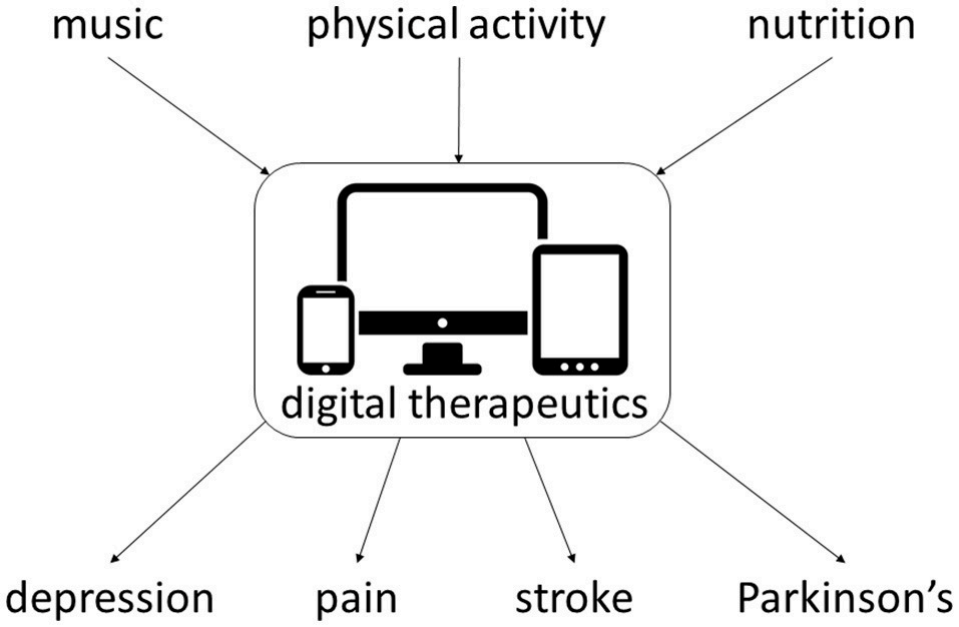
Examples of non-pharmacological interventions amenable for integration with digital therapeutics for chronic medical conditions. Multiple clinical studies support integration of such multimodal interventions for depression (Pirbaglou et al., 2016; Leubner and Hinterberger, 2017; Toups et al., 2017), pain (Cooney et al., 2013; Chai et al., 2017; Qaseem et al., 2017; Toups et al., 2017), stroke (Pollock et al., 2014a,b; Sihvonen et al., 2017), and Parkinson's disease (Seidl et al., 2014; Bloem et al., 2015; Roeder et al., 2015).

Digital therapeutics delivering multimodal interventions, including drug-device combination therapies, may benefit patients with neurological and other chronic medical conditions (Bulaj, 2014; Bulaj et al., 2016) including treatment of depression, pain, arthritis, and diabetes, given similar comorbidities. Music and mobile apps, such as SuperBetter (Roepke et al., 2015), MoodHacker (Birney et al., 2016), as well as web-based interventions (Merry et al., 2012; Buntrock et al., 2016) have been shown to reduce or prevent depressive symptoms (Firth et al., 2017; Leubner and Hinterberger, 2017), even in patients with refractory depression (Mantani et al., 2017). Virtual reality technologies improve pain relief and decrease opioid use (McSherry et al., 2017; Tashjian et al., 2017). Music-based interventions to manage acute and chronic pain may also show promise (Chai et al., 2017). Listening to the Mozart K.448 also produced beneficial clinical effects for people with schizophrenia taking antipsychotic drugs (He et al., 2018). These and other studies suggest that converting non-pharmacological interventions into mobile software as medical device and subsequent integration with antiseizure, antidepressant and analgesic medications is an attractive strategy for improving therapy outcomes in several neurologic disorders (Figure 7).

## Author contributions

PA, CB, MS, and GB conceived the project and defined digital content of the prototype mobile software for people with epilepsy; PA, CB, MS, GB, JJ, and DS designed the prototype mobile software; PA, CB, MS, and GB designed the survey questionnaire; PA, LF, FA, MG, MH, and KN collected survey results; PA, LF, and FA analyzed the survey results; PA, CB, MS, LF, FA, MG, MH, and KN reviewed the literature and discussed the results; PA, CB, MS, LF, FA, MG, MH, KN, JJ, and DS wrote and edited the manuscript.

### Conflict of interest statement

CB and GB are co-founders, officers and board members in Epicadence, Public Benefit Corporation, focused on development of mobile software for epilepsy patients. JJ is an officer in Epicadence, PBC, and a cofounder and an officer in Stretto Consulting. PA and MS are consultants to Epicadence, Public Benefit Corporation. PA, CB, MS, and GB are co-inventors on patent-pending “Multimodal Platform for Treating Epilepsy” licensed to Epicadence PBC. The patent claims describe methods and uses of coupling music and multimedia streaming with mobile app to reduce seizures in people with epilepsy. DS is a cofounder and an officer in WildOutWest. The other authors declare that the research was conducted in the absence of any commercial or financial relationships that could be construed as a potential conflict of interest.

## Abbreviations

HPA: the hypothalamic-pituitary-adrenal
K.448: Mozart's sonata K.448
PWE: people with epilepsy
SaMD: software as a medical device
UX: user experience.
