# Supplementary material for: Mobile Software as a Medical Device (SaMD) for the Treatment of Epilepsy: Development of Digital Therapeutics Comprising Behavioral and Music-Based Interventions for Neurological Disorders
Source: Front Hum Neurosci. 2018 May 1;12:171. doi: 10.3389/fnhum.2018.00171 (PMC5946004; doi:10.3389/fnhum.2018.00171)
Supplement: Supplementary file 1 [file Presentation_1.pdf]

Supplementary Material

Mobile Software as a Medical Device (SaMD) for the Treatment of Epilepsy: Development of Digital Therapeutics Comprising Behavioral and Music-based Interventions for Neurological Disorders

Pegah Afra, Carol S. Bruggers, Matthew Sweney, Lilly Fagatele, Fareeha Alavi, Michael Greenwald, Merodean Huntsman, Khanhly Nguyen, Jeremiah K. Jones, David Shantz, Grzegorz Bulaj

Corresponding Authors:
Pegah Afra: pegah.afra@hsc.utah.edu
Grzegorz Bulaj: bulaj@pharm.utah.edu

1. Supplementary data

The following are questions and answers from the questionnaire-based survey study among people with epilepsy:

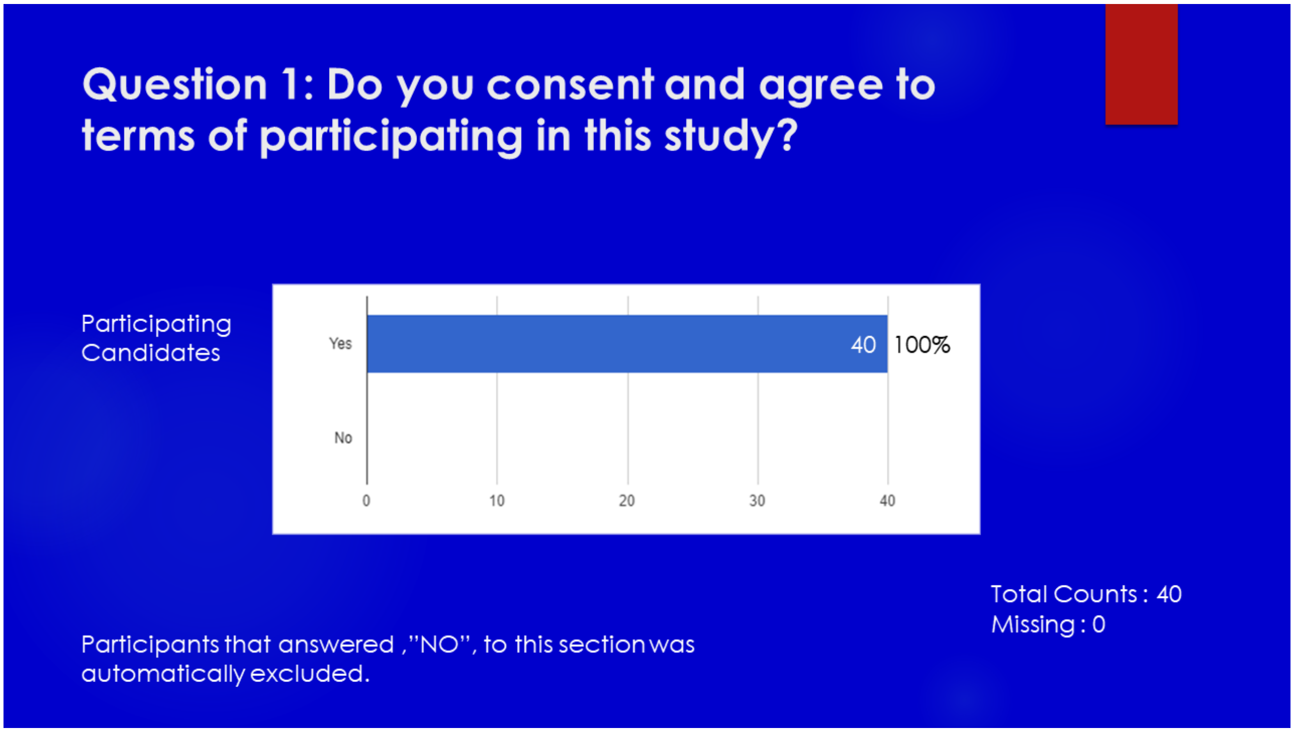

Question 2: Are you interested in using a mobile app to help manage your seizures ?

Accepting Candidates

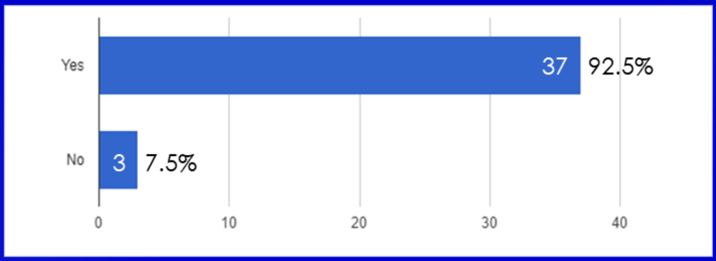

Total Counts : 40
Yes : 37
No : 3

Question 3: Which of the following feature(s) do you want in a mobile app?

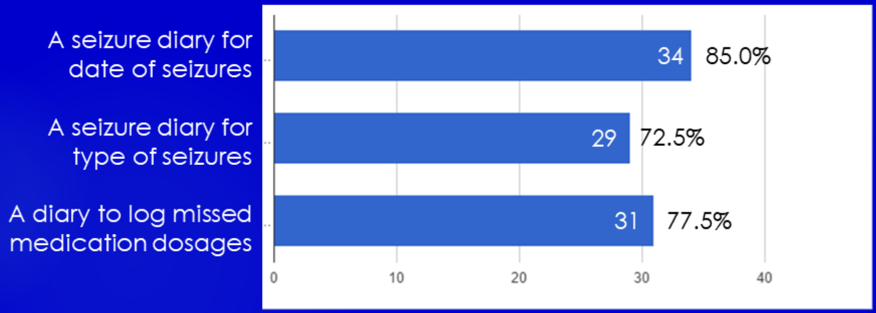

Total Counts : 40

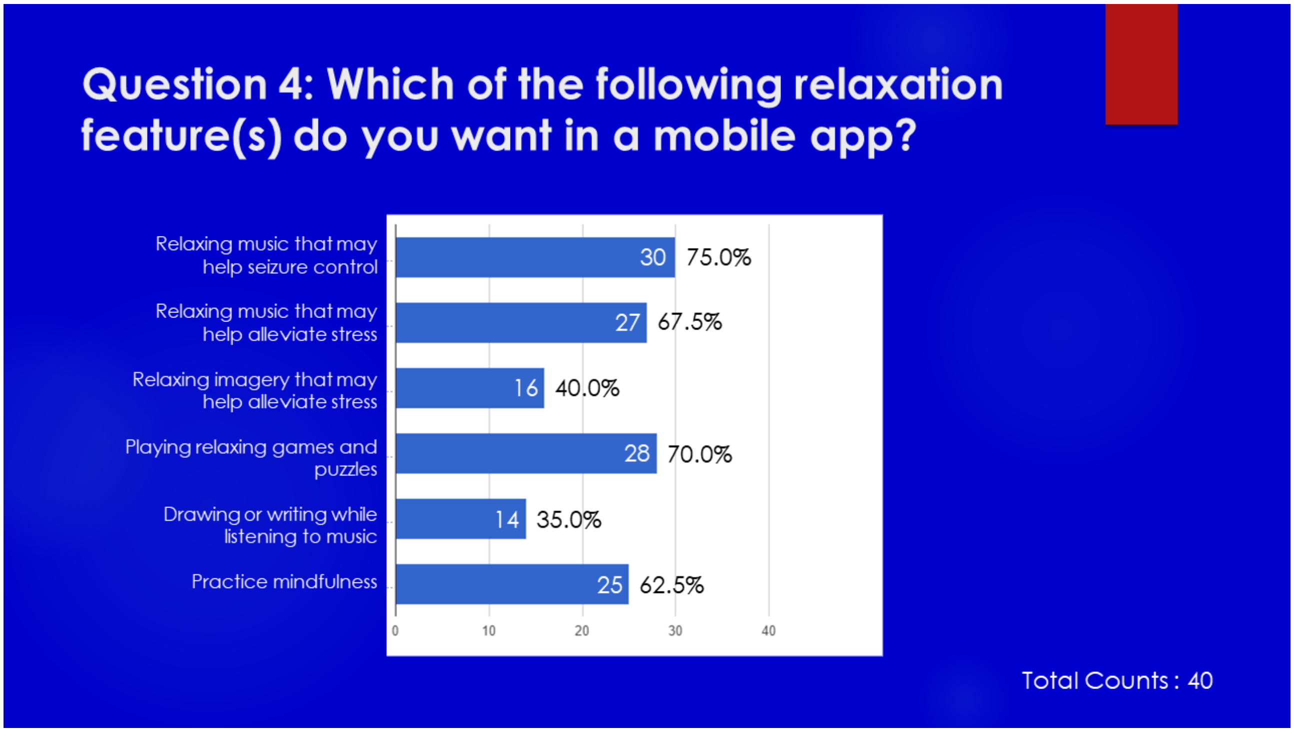
Question 4: Which of the following relaxation feature(s) do you want in a mobile app?
Relaxing music that may help seizure control
30
75.0%
Relaxing music that may help alleviate stress
27
67.5%
Relaxing imagery that may help alleviate stress
16
40.0%
Playing relaxing games and puzzles
28
70.0%
Drawing or writing while listening to music
14
35.0%
Practice mindfulness
25
62.5%
0
10
20
30
40
Total Counts : 40

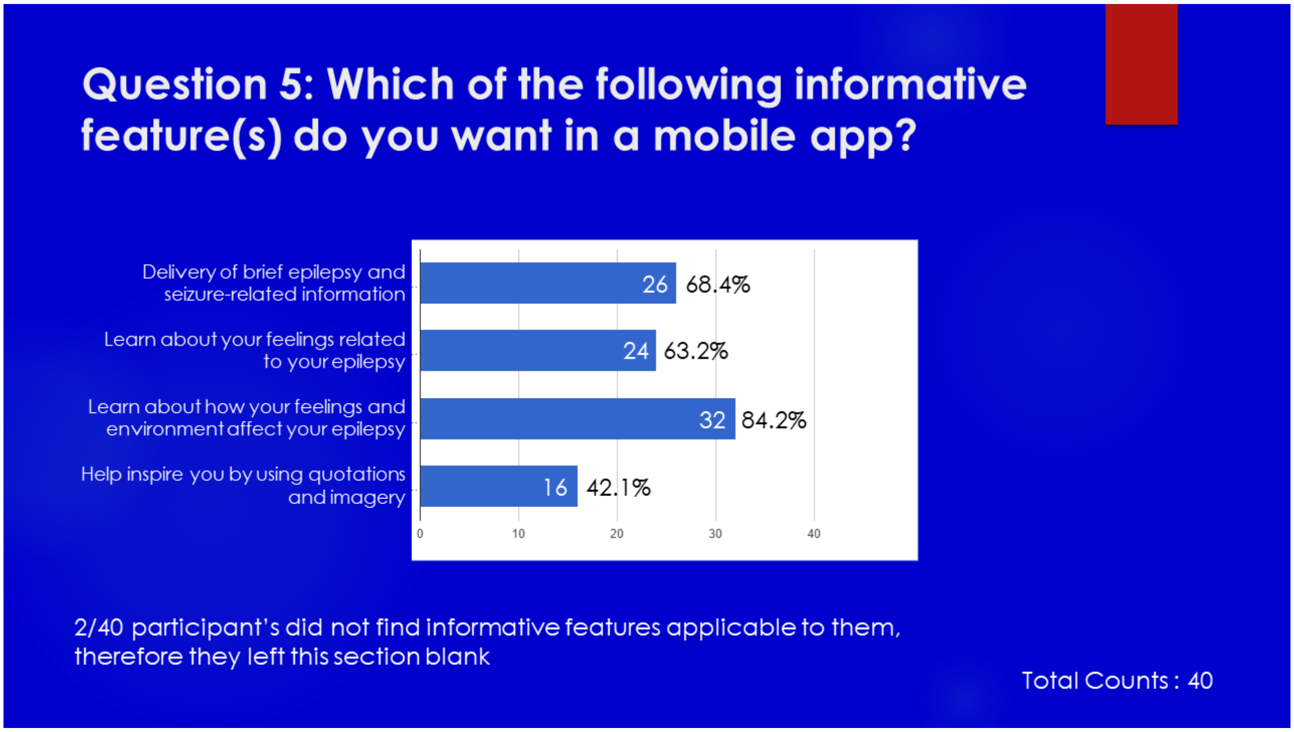
Question 5: Which of the following informative feature(s) do you want in a mobile app?
Delivery of brief epilepsy and seizure-related information
26
68.4%
Learn about your feelings related to your epilepsy
24
63.2%
Learn about how your feelings and environment affect your epilepsy
32
84.2%
Help inspire you by using quotations and imagery
16
42.1%
0
10
20
30
40
2/40 participant's did not find informative features applicable to them, therefore they left this section blank
Total Counts : 40

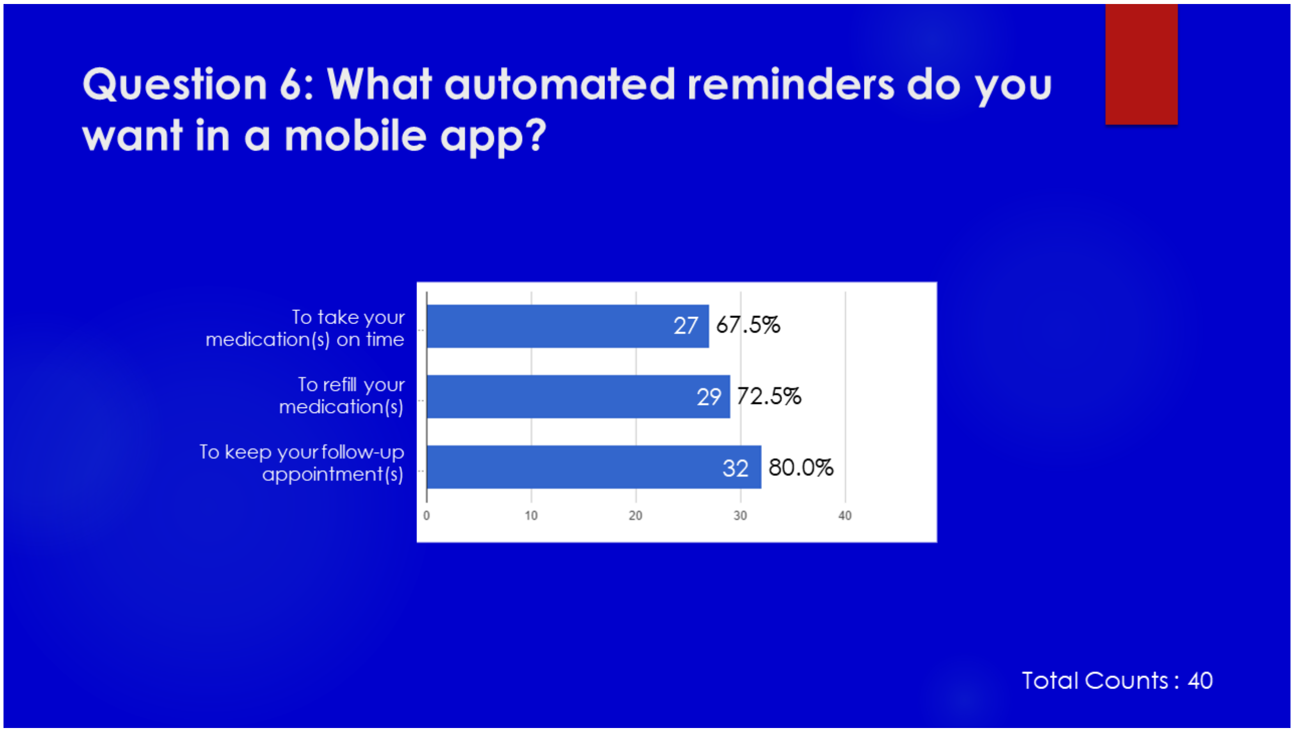
Question 6: What automated reminders do you want in a mobile app?
To take your medication(s) on time
27
67.5%
To refill your medication(s)
29
72.5%
To keep your follow-up appointment(s)
32
80.0%
0
10
20
30
40
Total Counts : 40

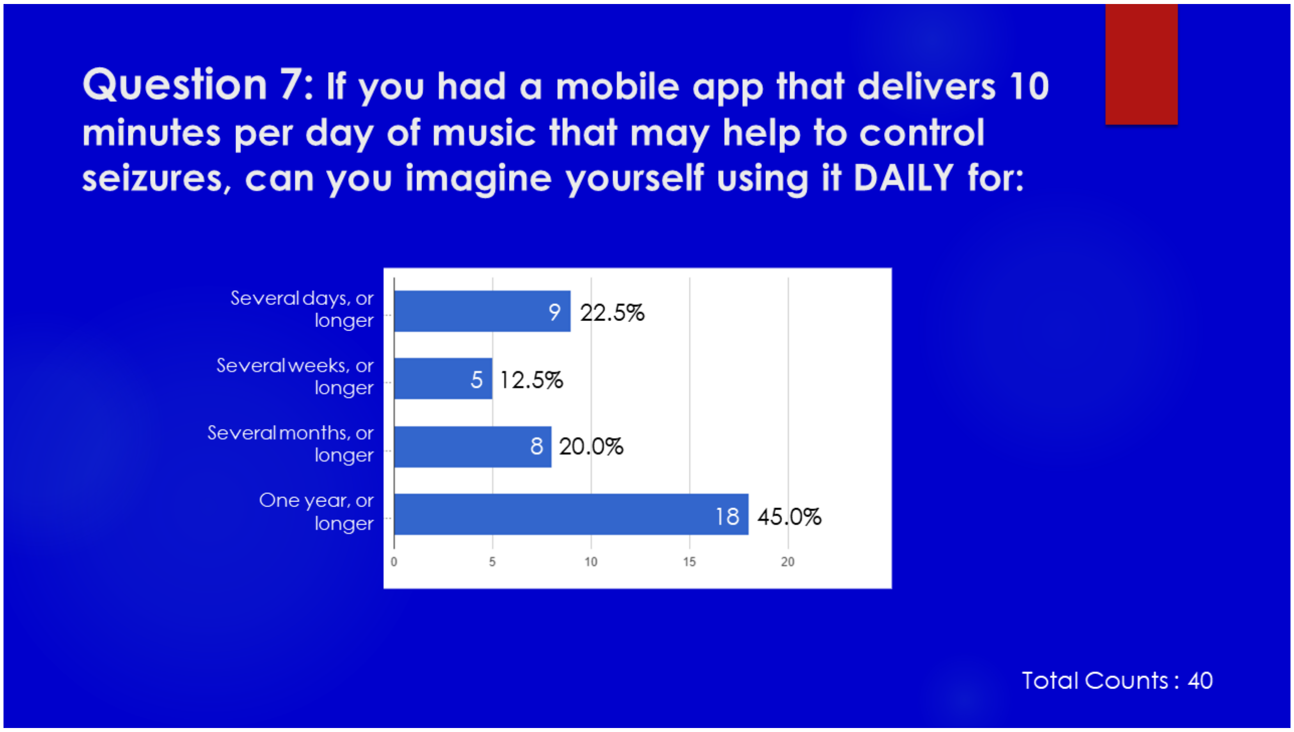
Question 7: If you had a mobile app that delivers 10 minutes per day of music that may help to control seizures, can you imagine yourself using it DAILY for:
Several days, or longer
9
22.5%
Several weeks, or longer
5
12.5%
Several months, or longer
8
20.0%
One year, or longer
18
45.0%
0
5
10
15
20
Total Counts : 40
